# Low referral uptake, not patient adherence, limits implementation of early nutritional screening in head and neck and upper gastrointestinal cancers

**DOI:** 10.3389/fnut.2026.1817590

**Published:** 2026-08-03

**Authors:** Irene Azzali, Debora Guerra, Monia Suzzi, Alessandro Passardi, Sebastiano Calpona, Ilario Giovanni Rapposelli, Margherita Muratore, Francesco De Rosa, Elisabetta Parisi, Roberta Volpi, Patrizia Serra

**Affiliations:** IRCCS Istituto Romagnolo per lo Studio dei Tumori (IRST) “Dino Amadori”, Meldola, Italy

**Keywords:** head and neck cancer, implementation, malnutrition, nutritional screening, supportive care

## Abstract

**Background:**

Malnutrition is highly prevalent in patients with head and neck and upper gastrointestinal cancers and is associated with poorer clinical outcomes. Despite guideline recommendations, early nutritional screening remains inconsistently implemented in routine oncology care.

**Methods:**

This single-center prospective feasibility study evaluated the implementation of an early nutritional screening and intervention pathway in newly diagnosed oncology patients with head and neck and upper gatrointestinal cancers. Eligible patients were enrolled within 7 days of their first oncology consultation and entered a structured nutritional pathway including baseline (T0), one-month (T1), and two-month (T2) assessments. Nutritional evaluation included MUST screening, anthropometric measures, bioelectrical impedance analysis, handgrip strength, biochemical parameters, and patient-reported outcomes (ESAS and FACT-G). Feasibility endpoints included referral, enrolment, and adherence rates.

**Results:**

Among 550 eligible patients, 77 were enrolled (14%). Once enrolled, adherence to the pathway was high (91%). At baseline, 47% of patients were classified as at high nutritional risk. Serum biochemical markers, available in a subset of patients, were consistent with mild nutritional alterations. During follow-up, body weight, handgrip strenght, biochemical and bioelectrical impedance parameters, and overall quality of life remained stable. Significant improvements were observed in selected symptoms, including fatigue, nausea, and well-being/malaise.

**Conclusion:**

Early nutritional screening is feasible and highly acceptable once implemented. However, implementation at the population level is limited by low and non-systematic referral rates, highlighting a critical gap in routine oncology care.

## 1 Background

Patients with cancers of the head and neck and upper gastrointestinal (GI) tract are at particularly high risk of developing malnutrition due to the tumor location, treatment-related toxicities, and cancer-associated metabolic alterations. Head and neck cancers include malignancies of the oral cavity, oropharynx, and larynx. These tumors are frequently associated with dysphagia, odynophagia, xerostomia, and reduced oral intake, which contribute to early and progressive nutritional decline. Upper GI cancers, including esophageal, gastric, pancreatic, biliary tract, and liver cancers, are more commonly characterized by malabsorption, pancreatic exocrine insufficiency, nausea, early satiety, and cancer-related cachexia.

Treatment of both disease groups typically involves surgery, radiotherapy, chemotherapy, or multimodal approaches. In head and neck cancers, chemoradiotherapy is often associated with mucositis and severe swallowing impairment, while surgical interventions and systemic treatments in upper GI cancers may result in prolonged nutritional compromise due to anatomical and functional alterations.

Malnutrition remains a frequent but under-recognized complication in oncology, with reported prevalence exceeding 60–80% in these patient populations. It is associated with increased treatment toxicity, postoperative complications, infection rates, hospitalizations, functional decline, and reduced quality of life (QoL), and it negatively impacts survival outcomes (1–6).

Despite strong recommendations from ESPEN and AIOM–SINPE guidelines for early nutritional screening in all oncology patients (7–12), implementation in routine clinical practice remains inconsistent. This gap is partly due to organizational and systemic barriers, leading to delayed identification of at-risk patients and missed opportunities for early intervention (13–15).

In this context, the ENutriScreen (IRST 100.55) study was designed as a single-center, prospective pilot study to evaluate the implementation of an early nutritional screening and intervention pathway (16, 17) in patients with newly diagnosed head and neck and upper GI cancers.

The primary aim was to assess feasibility of integrating structured nutritional care into routine oncology prac7tice, while secondary exploratory analyses evaluated its potential clinical impact using nutritional and patients-reported outcomes.

## 2 Materials and methods

### 2.1 Study population

ENutriScreen (IRST 100.55) is a single-center, prospective observational study conducted at the IRCCS Istituto Romagnolo per lo Studio dei Tumori (IRST), aimed at evaluating the feasibility and organizational implementation of early nutritional screening pathway in newly diagnosed oncology patients.

Patients were eligible if they were ≥ 18 years older, had a histologically confirmed diagnosis of head and neck or upper GI cancer, and had undergone their first oncology consultation within the previous 7 days. Estimated life expectancy of at least 3 months was required, based on clinical judgment and Eastern Cooperative Oncology Group (ECOG) performance status. Patients received standard oncological management according to tumor type, disease stage, and multidisciplinary evaluation, including surgery, chemotherapy, radiotherapy, or combined therapeutic approaches, when clinically indicated. All participants provided written informed consent. The study was approved by the Regional Ethics Committee of Romagna (CEROM) and conducted in accordance with the Declaration of Helsinki and ICH-GCP guidelines.

### 2.2 Nutritional screening

During the study period, nutritional referral was performed during the first oncological visit based on the treating oncologist’s subjective clinical assessment of nutritional status and risk. Patients considered at nutritional risk or presenting signs suggestive of malnutrition were referred to the Nutrition Outpatient Clinic for further evaluation. Within 7 days of their initial oncology visit, eligible patients were enrolled to enter a structured nutritional screening and follow-up pathway, including assessment at baseline (T0), 1 month (T1), and 2 months (T2). At each visit, patients underwent nutritional risk screening with the Malnutrition Universal Screening Tool (MUST) (18, 19), anthropometric assessment (weight, height, BMI, and circumferences), bioimpedance analysis (phase angle, body cell mass, and hydration status) and hand grip strength measurement using bilateral dynamometry (20–25). Biochemical parameters (albumin, prealbumin, transferrin, vitamin D, vitamin B12, folates, and lipid profile) were collected when available as part of routine clinical care (26, 27). Patient-reported outcomes included the Edmonton Symptom Assessment Scale (ESAS) (28) and the Functional Assessment of Cancer Therapy-General (FACT-G) (29). Nutritional management was individualized and included dietary counseling alone, oral nutritional supplementation (ONS), or referral for artificial nutrition (enteral or parenteral support), depending on clinical need and nutritional risk. Dietary intake assessment (24-h recall and a food diary) was collected when available in routine practice. These assessments were repeated at each scheduled visit. Adherence to nutritional prescriptions and the need for modifications were documented throughout the study. Patients reporting severe anorexia or gastrointestinal symptoms received individualized dietary modifications, including texture-adapted meals and antiemetic strategies. Oral nutritional supplements were prescribed in cases of inadequate intake, and artificial nutrition was initiated when clinically indicated, in collaboration with regional dietetic services. Continuity of care was ensured through scheduled follow-up visits and telephone contacts.

### 2.3 Feasibility endpoints

The primary objective was to assess feasibility of implementing the nutritional pathway. Feasibility was evaluated using referral rate (proportion of eligible patients referred by the oncologist to the nutritional pathway), consent (enrolment) rate (proportion of referred patients who accepted participation and attended the baseline visit) and adherence rate (proportion of enrolled patients completing all scheduled visit T0-T2). Predefined feasibility thresholds were ≥70% for referral/enrolment and ≥80% for adherence, based on supportive care implementation studies.

### 2.4 Data collection

Patient data were collected using paper-based and electronic case report forms. Baseline characteristics included age, sex, tumor site, stage (TNM), BMI, treatment modality, and nutritional risk indicators. BIA was performed using a standardized protocol (Akern BIA 101 Anniversary) and analyzed with Body Gram Plus software.

### 2.5 Statistical analysis

Given the exploratory nature of this feasibility study, no formal sample-size calculation was performed. Continuous variables were reported as mean ± standard deviation (sd) or median and interquartile range (IQR), first quartile-Q1 and third quartile-Q3), as appropriate. Categorical variables were reported as frequencies and percentages. Moderate–severe symptoms of ESAS were defined as scores ≥4 (range 0–10). Total FACT-G score was calculated as the sum of the four subscale scores. Missing questionnaire items were handled using domain mean imputation when ≥50% of items were completed. Longitudinal changes across time points (T0, T1, T2) were analyzed using non-parametric repeated-measures ANOVA (nparLD package in R). Statistical analyses were performed using STATA version 15.0 (StataCorp, Texas, USA).

## 3 Results

### 3.1 Study feasibility

During the enrollment period (February 2022–February 2024), 550 patients were identified as eligible, of whom 77 underwent baseline nutritional screening and 473 were not referred to the nutritional pathway. Among the 473 eligible patients who were not referred, median age was 70 years (IQR 60–78) and 60% were male. ECOG performance status was 0 in 63%, 1 in 21%, 2 in 10%, and 3 in 5%. Most non-referred patients had upper gastrointestinal tumors (92%), while 8% had head and neck cancers. The 77 patients who underwent baseline nutritional screening corresponded to an overall consent rate (CR) of 14%, which did not reach the predefined feasibility threshold of 70%. When stratified by tumor site, patients with head and neck cancers had a markedly higher CR (22 of 44, 50%) compared with those with upper GI tumors (55 of 506, 11%). Despite the low CR, once enrolled, adherence to the nutritional pathway was high: 70 of 77 patients (91%) completed all three scheduled visits (T0, T1, T2), exceeding the predefined adherence criterion of 80%. Targeted interventions were delivered according to baseline nutritional status and symptom burden.

### 3.2 Patient characteristics

Baseline demographic and clinical features are summarized in Table 1. The cohort included 77 eligible patients; however, complete data were available for 68 patients for some study variables due to missing or incomplete assessments. Of the 77 patients, 52 were males (68%) and 25 were females (32%), with a median age of 68 years (IQR 57–72). The most frequent tumor sites were pancreas (38%), oral cavity (18%), stomach (16%) and esophagus (10%). Disease stage distribution was stage I in 9% of patienst, stage II in 26%, stage III in 21% and stage IV in 44%. Regarding anticancer treatment, 47 patients (61% were candidates for chemotherapy, 20 (26%) for chemoradiotherapy, and 9 (12%) for radiotherapy alone. Overall, 56 patients (73%) had previously received oral nutritional supplements (ONS), although only 5% were still receiving ONS at study entry; similarly, only 5% had previously undergone a nutritional evaluation. Enteral nutrition was administered in 2 patients (3%), while 16 (21%) were receiving parenteral nutrition. According to body mass index (BMI) categories, 9% of patients were underweight, 45% had normal weight, 33% were overweight, and 13% were obese. Based on the MUST classification, 47% of patients were at high nutritional risk (score ≥2), while 20% were at moderate risk (score = 1). Functional parameters confirmed early nutritional impairment. Median handgrip strength was 32.4 kg (IQR 23.9–39.2) in the right hand and 31.9 kg (IQR 26.1–37.6) in the left hand. Baseline biochemical parameters, available in a subset of patients, showed mild alterations consistent with early nutritional impairment. Reduced prealbumin and folic acid levels were the most frequent abnormalities, observed in 40 and 43% of evaluable patients, respectively, while low transferrin levels were detected in 26% of cases (Table 2). Most patients had normal serum albumin, total protein, vitamin B12, and vitamin D levels, whereas elevated gamma-glutamyl transferase and glucose values were frequently observed, particularly among patients with upper GI cancers. Bioelectrical impedance analysis was reported in Table 3.

**Table 1:** Patient characteristics.

	Overall (*n* = 77)	Head & neck cancers (*n* = 22)	Upper gastrointestinal cancers (*n* = 55)
Age			
Mean, sd	65, 11	65, 12	64, 11
Median, Q1-Q3	68, 57–72	66, 54–75	68, 57–72
Sex – n (%)			
Female	25 (32)	4 (18)	21 (38)
Male	52 (68)	18 (82)	34 (62)
Tumor site – n (%)			
Oral cavity	14 (18)	14 (64)	-
Oropharynx	5 (6)	5 (23)	-
Larynx	3 (4)	3 (13)	-
Pancreas	29 (38)	-	29 (53)
Stomach	12 (16)	-	12 (22)
Esophagus	8 (10)	-	8 (14)
Biliary tract	5 (6)	-	5 (9)
Liver	1 (1)	-	1 (2)
Stage – n (%)			
I	7 (9)	1 (5)	6 (11)
II	20 (26)	6 (27)	14 (25)
III	16 (21)	7 (32)	9 (16)
IV	34 (44)	8 (36)	26 (48)
Previous treatment performed – n (%)			
Surgery	10 (13)	4 (18)	6 (11)
None	67 (87)	18 (82)	49 (89)
Previous evaluation at the nutritional service – n (%)			
Yes	4 (5)	3 (14)	1 (2)
No	73 (95)	19 (76)	54 (98)
Nutritional support – n (%)			
Oral nutritional supplements	56 (73)	19 (86)	37 (67)
No	21 (27)	3 (14)	18 (33)
Type of nutrition – n (%)			
Enteral	2 (3)	0	2 (4)
Parenteral	16 (21)	8 (36)	8 (15)
BMI – n (%)			
Underweight	7 (9)	1 (5)	6 (11)
Normal weight	34 (45)	8 (36)	27 (50)
Overweight	25 (33)	11 (50)	13 (24)
Obese	10 (13)	2 (9)	8 (15)
Missing	1	0	1
MUST score – n (%)			
0	26 (34)	12 (55)	14 (26)
1	15 (20)	4 (18)	11 (20)
≥2	35 (47)	6 (27)	29 (54)
2	24	4	20
3	6	1	5
4	5	1	4
Missing	1	0	1
Hand grip strength-right kg			
Mean, sd	33.8, 15.9	36.3, 10.5	32.9, 17.7
Median, Q1-Q3	32.4, 23.9–39.2	33.7, 30.0–40.0	29.9, 20.8–38.6
Missing	4	1	3
Hand grip strength-left kg			
Mean, sd	33.4, 14.2	37.5, 10.1	31.8, 15.4
Median, Q1-Q3	31.9, 26.1–37.6	34.6, 31.0–43.8	31.1, 22.3–35.7
Missing	7	1	6

sd, standard deviation; Q1-Q3, First and Third quartile; BMI, body mass index; MUST, Malnutrition Universal Screening Tool.

**Table 2:** Biochemical parameters.

	Overall (*n* = 77)	Head & Neck cancers (*n* = 22)	Upper gastrointestinal cancers (*n* = 55)
Total proteins (g/L) – n (%)			
<Lower normal limit	6 (13)	3 (23)	3 (9)
Normal limit	39 (85)	10 (77)	29 (88)
>Upper normal limit	1 (2)	0	1 (3)
Missing	31	9	22
Albumin (g/L) – n (%)			
<Lower normal limit	4 (8)	0	4 (11)
Normal limit	45 (90)	13 (100)	32 (86)
>Upper normal limit	1 (2)	0	1 (3)
Missing	27	9	18
Prealbumin (g/L) – n (%)			
<Lower normal limit	20 (40)	3 (23)	17 (46)
Normal limit	30 (60)	10 (77)	20 (54)
>Upper normal limit	0	-	-
Missing	27	9	18
Transferrin (g/L) – n (%)			
<Lower normal limit	13 (26)	3 (23)	10 (27)
Normal limit	37 (74)	10 (77)	27 (73)
>Upper normal limit	0	-	-
Missing	27	9	18
Vitamin B12 (pmol/L) – n (%)			
<Lower normal limit	1 (2)	0	1 (3)
Normal limit	45 (90)	12 (92)	33 (89)
>Upper normal limit	4 (8)	1 (8)	3 (8)
Missing	27	9	18
Folic acid (nmol/L) – n (%)			
<Lower normal limit	20 (43)	4 (40)	16 (44)
Normal limit	26 (57)	6 (60)	20 (56)
>Upper normal limit	0	-	-
Missing	31	12	19
Vitamin D (ug/L) – n (%)			
<Lower normal limit	7 (15)	0	7 (21)
Normal limit	39 (85)	13 (100)	26 (79)
Missing	31	9	22
Gamma-glutamyl transferase (U/L) – n (%)			
<Lower normal limit	1 (1)	0	1 (2)
Normal limit	37 (58)	11 (79)	26 (52)
>Upper normal limit	26 (41)	3 (21)	23 (46)
Missing	13	8	5
Glucose (mg/dl) – n (%)			
<Lower normal limit	0	-	-
Normal limit	36 (55)	8 (57)	28 (55)
>Upper normal limit	29 (45)	6 (43)	23 (45)
Missing	12	8	4
Total cholesterol (mg/dl) – n (%)			
Normal limit	49 (75)	8 (57)	41 (80)
>Upper normal limit	16 (25)	6 (43)	10 (20)
Missing	12	8	4
LDL cholesterol (mg/dl) – n (%)			
Normal limit	48 (74)	8 (57)	40 (78)
>Upper normal limit	17 (26)	6 (43)	11 (22)
Missing	12	8	4
HDL cholesterol (mg/dl) – n (%)			
Normal limit	19 (29)	0	19 (37)
>Upper normal limit	46 (71)	14 (100)	32 (63)
Missing	12	8	4
Triglycerides (mg/dl) – n (%)			
Normal limit	55 (85)	12 (86)	43 (84)
>Upper normal limit	10 (15)	2 (14)	8 (16)
Missing	12	8	4

**Table 3:** Bioimpedance parameters.

	Overall (*n* = 77)	Head & Neck cancers (*n* = 22)	Upper gastrointestinal cancers (*n* = 55)
RZ (ohm)			
Mean, sd	498.3, 82.97	484.5, 64.73	504.0, 89.38
Median, Q1-Q3	496.0, 441.0–549.0	474.0, 448.0–502.0	510.0, 433.0–566.0
Missing	5	1	4
XC (ohm)			
Mean, sd	49.2, 9.91	48.7, 11.25	49.5, 9.41
Median, Q1-Q3	50.5, 40.5–57.0	47.0,39.0–57.0	51.0, 42.0–57.0
Missing	5	1	4
PhA			
Mean, sd	5.7, 1.07	5.7, 1.50	5.7, 1.07
Median, Q1-Q3	5.7, 4.95–6.2	5.7, 5.3–6.2	5.6, 4.9–6.2
Missing	5	1	4
BCMI			
Mean, sd	11.4, 11.88	14.9, 21.63	10.0, 2.45
Median, Q1-Q3	9.7, 8.7–11.5	10.6, 9.4–11.8	9.5, 8.2–11.4
Missing Missing	5	1	4
BMR (Kcal)			
Mean, sd	1588.1, 235.38	1613.8, 169.01	1577.5, 258.57
median, Q1-Q3	1604.0, 1401.0–1739.0	1647.0, 1539.0–1719.0	1573.0, 1388.0–1745.0
missing	5	1	4
BCM (kg)			
Mean, sd	28.9, 8.10	29.8, 5.82	28.5, 8.90
Median, Q1-Q3	29.5, 22.5–34.1	30.9, 27.2–33.4	28.4, 22.0–34.3
Missing	5	1	4
BCM (%)			
Mean, sd	51.7, 6.64	52.0, 5.65	51.6, 5.70
Median, Q1-Q3	52.0, 48.0–54.8	52.3, 50.1–54.9	51.7, 47.5–54.7
Missing	5	1	4
FFM (kg)			
Mean, sd	55.3, 11.78	57.1, 8.39	54.6, 12.92
Median, Q1-Q3	56.7, 46.1–62.3	58.2, 51.0–62.4	54.5, 41.8–61.7
Missing	5	1	4
FFM (%)			
Mean, sd	79.4, 10.76	79.3, 7.19	79.4, 11.99
Median, Q1-Q3	80.8, 73.3–86.1	78.5, 72.2–84.0	83.4, 73.8–86.7
Missing	5	1	4
FM (kg)			
Mean, sd	14.6, 8.63	15.6, 6.89	14.1, 9.28
Median, Q1-Q3	12.6, 8.5–19.5	15.3, 10.3–20.5	10.7, 7.7–19.4
Missing	5	1	4
FM (%)			
Mean, sd	19.8, 8.18	20.7, 7.19	19.4, 8.59
Median, Q1-Q3	19.1, 13.8–26.2	21.5, 16.0–27.8	16.6, 13.3–26.2
Missing	5	1	4
TBW (L)			
Mean, sd	41.1, 8.98	42.4, 6.09	40.5, 9.93
Median, Q1-Q3	41.6, 34.1–46.1	42.7, 39.8–46.2	41.5, 30.9–46.0
Missing	5	1	4
TBW (%)			
Mean, sd	59.5, 6.23	58.8, 5.52	59.7, 6.53
Median, Q1-Q3	60.2, 54.5–63.8	57.9, 54.2–61.2	61.3, 55.2–64.0
Missing	5	1	4
ECW (L)			
Mean, sd	19.4, 4.10	20.1, 3.46	19.1, 4.34
Median, Q1-Q3	18.6, 16.0–22.3	20.4, 17.7–23.1	18.3, 15.7–21.5
Missing	5	1	4
ECW (%)			
Mean, sd	47.1, 7.48	45.5, 11.18	47.7, 5.29
Median, Q1-Q3	47.3, 44.7–51.1	47.0, 44.6–49.1	47.6, 44.8–51.5
Missing	5	1	4
Nutrigram			
Mean, sd	818.7, 289.37	895.0, 172.18	787.3, 321.87
Median, Q1-Q3	861.1, 634.1–1125.9	914.3, 828.5–991.4	793.7, 579.4–984.5
Missing	5	1	4
Idragram			
Mean, sd	124.0, 213.79	74.2, 2.08	144.9, 252.56
Median, Q1-Q3	73.5, 73.1–73.9	73.6, 73.1–73.9	73.4, 73.1–73.9
Missing	6	1	5

sd, standard deviation; Q1-Q3, First and Third quartile; RZ, resistance; XC, reactance; PhA, phase angle; BCMI, body cell mass index; BMR, basal metabolic rate; BCM, body cell mass; FFM, fat-free mass; FM, fat mass; TBW, total body water; ECW, extracellular water.

### 3.3 Symptom burden and quality of life at baseline

At the initial nutritional consultation (T0), 68 patients (88%) completed the ESAS questionnaire (Table 4). The most frequent symptoms at moderate-to-severe intensity (score ≥4) were fatigue (54%), malaise (49%), drowsiness and anxiety (40%), and loss of appetite (38%). Median (IQR) scores were 4 (1–7) for fatigue, 3 (1–5) for malaise, 2 (0–8) for drowsiness, 2 (0–10) for anxiety, and 3 (0–6) for appetite loss. Gastrointestinal symptoms such as nausea [0 (0–1)] and diarrhea [0 (0–0)] were generally mild, with ≥85% of patients reporting scores <4. Overall distress, assessed by the Total Distress Score (TDS), had a median of 15 (IQR 4–28); 73% of patients fell within the mild category (0–24), 25% in the moderate category (25–48), and 3% in the severe category (49–80). Quality of life, assessed by the FACT-G questionnaire (Table 5), showed a mean total score of 74 ± 17. Domain analysis revealed lower values for functional (mean 14 ± 7) and emotional well-being (16 ± 5), intermediate scores for social well-being (21 ± 6), whereas physical well-being was relatively preserved (23 ± 5). When categorized, 6% of patients were in the low QoL group (0–45), 46% in the moderate QoL group (46–75) and 48% in the high QoL range (≥76).

**Table 4:** Baseline symptom burden (ESAS).

	Completed ESAS T0 (*n* = 68)		
	Overall	Head & Neck cancers	Upper gastrointestinal cancers
Pain			
Mean, sd	2, 3	2, 3	3, 3
Median, Q1-Q3	2, 0–4	1, 0–4	2, 0–8
Moderate–Severe – n (%)	22 (32)	6 (30)	16 (67)
Fatigue			
Mean, sd	4, 3	4, 3	4, 3
Median, Q1-Q3	4, 1–7	4, 0–5	4, 1–7
Moderate–Severe – n (%)	37 (54)	11 (55)	26 (54)
Nausea			
Mean, sd	1, 2	0, 0	1, 2
Median, Q1-Q3	0, 0–1	0, 0–0	0, 0–2
Moderate–Severe – n (%)	8 (12)	0	8 (17)
Depression			
Mean, sd	2, 3	2, 3	2, 3
Median, Q1-Q3	0.5, 0–5	0, 0–5	1, 0–5
Moderate–Severe – n (%)	21 (31)	7 (35)	14 (29)
Anxiety			
Mean, sd	3, 3	3, 3	3, 3
Median, Q1-Q3	2, 0–6	1, 0–4	3, 0–6
Moderate–Severe – n (%)	27 (40)	6 (30)	21 (44)
Appetite loss			
Mean, sd	3, 3	2, 2	4, 3
Median, Q1-Q3	3, 0–6	2, 0–3	3, 0–6
Moderate–Severe – n (%)	26 (38)	4 (20)	22 (46)
Malaise			
Mean, sd	4, 3	3, 3	4, 3
Median, Q1-Q3	3, 1–5	4, 0–5	3, 1–5
Moderate–Severe – n (%)	33 (49)	11 (55)	22 (46)
Dyspnea			
Mean, sd	1, 2	1, 2	1, 2
Median, Q1-Q3	0, 0–1	0, 0–2	0, 0–1
Moderate–Severe – n (%)	9 (13)	4 (20)	5 (10)
Drowsiness			
Mean, sd	3, 3	2, 3	3, 3
Median, Q1-Q3	2, 0–5	1, 0–4	2, 0–5
Moderate–Severe – n (%)	27 (40)	6 (30)	21 (44)
Total distress score			
Mean, sd	17, 15	16, 12	19, 15
Median, Q1-Q3	15, 4–28	14, 4–22	17, 6–28

sd, standard deviation; Q1-Q3, First and Third quartile.

**Table 5:** Baseline quality of life domains (FACT-G).

	Completed FACT-G T0 (*n* = 68)		
	Overall	Head & Neck cancers	Upper gastrointestinal cancers
Physical well-being			
Mean, sd	23, 5	25, 3	22, 5
Median, Q1-Q3	25, 20–27	25, 22–28	24, 18–26
Social/family well-being			
Mean, sd	21, 6	21, 6	20, 5
Median, Q1-Q3	21, 17–25	22.5, 18–25.5	21, 17–23
Emotional well-being			
Mean, sd	16, 5	18, 4	15, 5
Median, Q1-Q3	17.5, 13–19	19, 17–21	15, 12–19
Functional well-being			
Mean, sd	14, 7	14, 8	14, 7
Median, Q1-Q3	12.5, 8–20	15, 8–20	12, 7–19
Total FACT-G score			
Mean, sd	74, 17	79, 15	71, 17
Median, Q1-Q3	75, 60–89	81, 68–91	71, 53–87

FACT-G, Functional Assessment of Cancer Therapy-General; sd, standard deviation; Q1–Q3, First and Third quartile.

### 3.4 Exploratory efficacy outcomes

**Figure 1:**
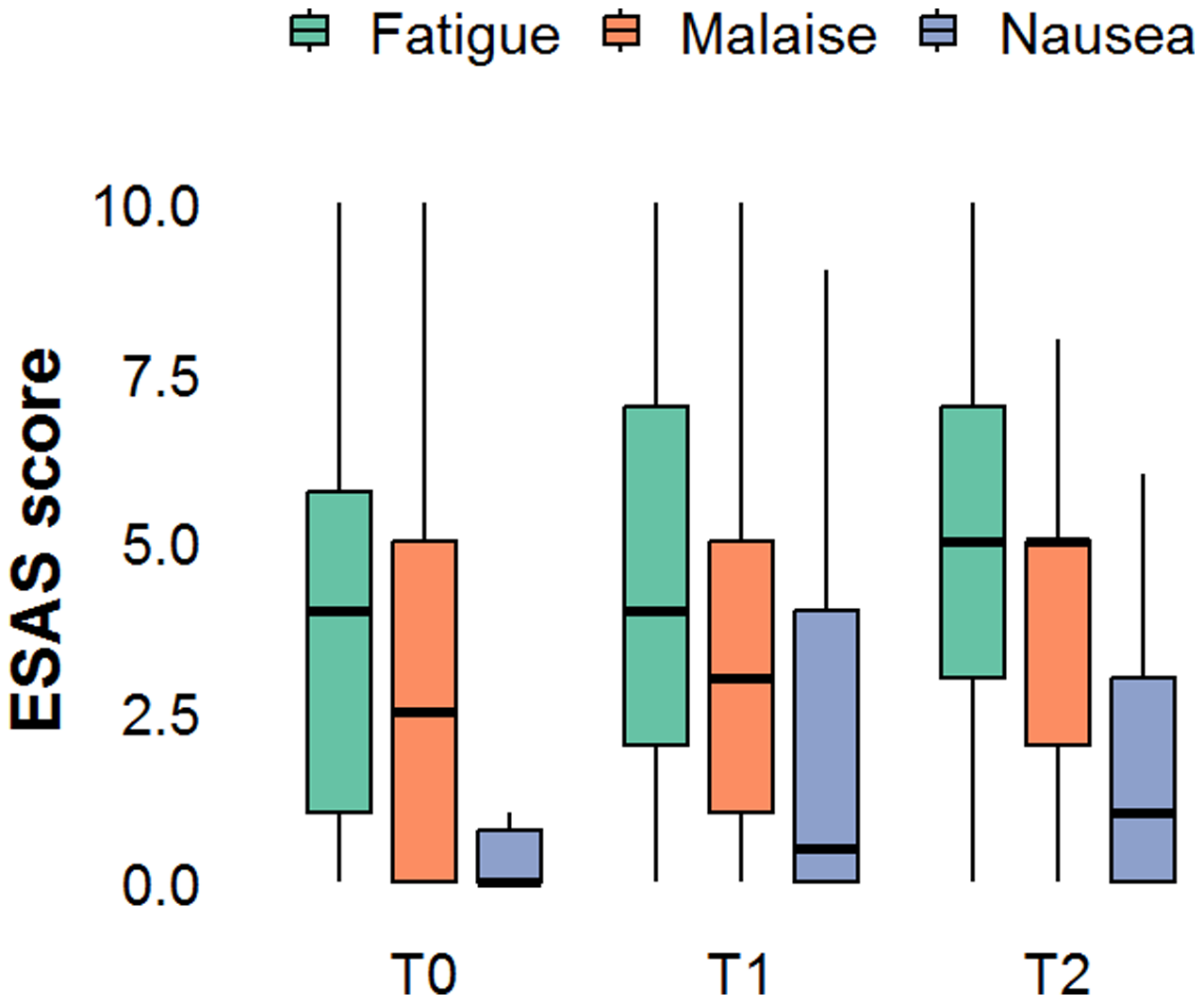
Boxplot of ESAS items over time (fatigue, malaise, nausea).

**Figure 2:**
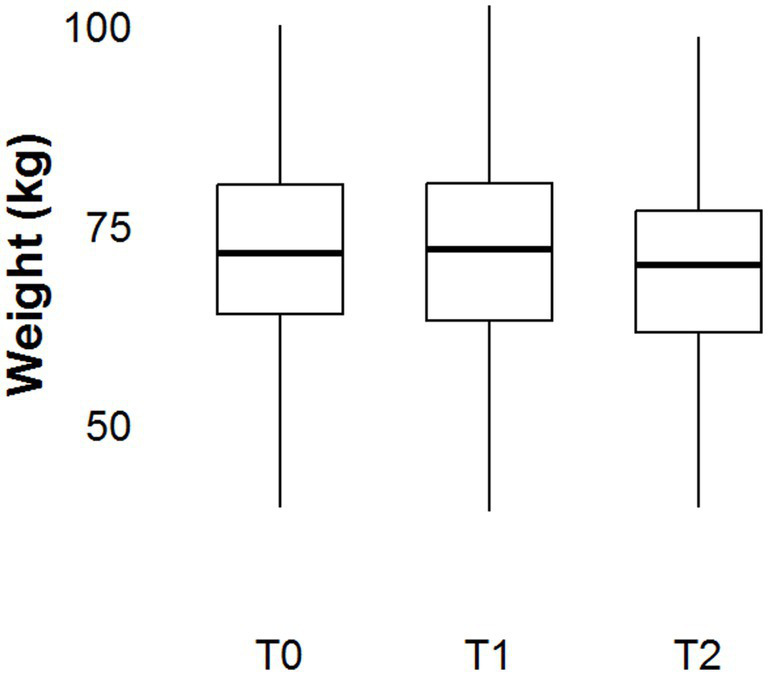
Boxplot of weight over time.

**Figure 3:**
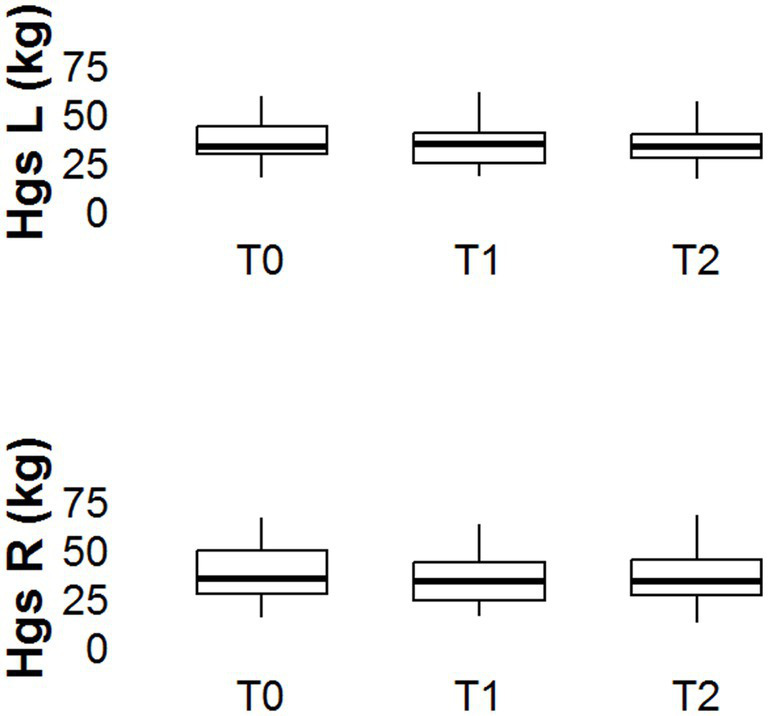
Boxplot of handgrip strength over time.

**Figure 4:**
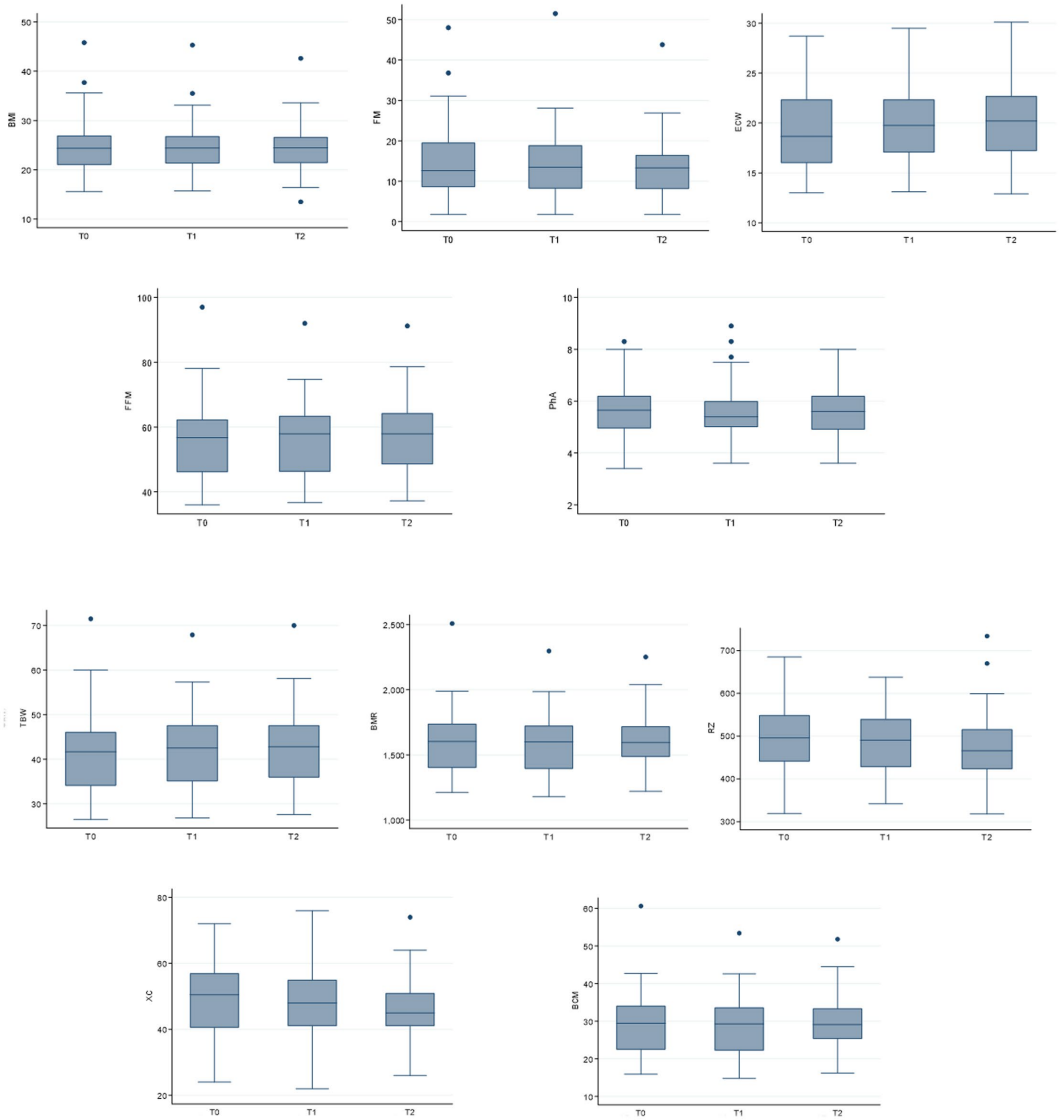
Boxplot of bioimpedance parameters over time.

For patients who completed the nutritional plan, exploratory analyses of secondary efficacy indicators were performed over time. Quality of life (FACT-G) remained stable: mean total score was 74 ± 17 at baseline, 75 ± 17 at T1, and 73 ± 14 at T2; no significant differences were observed (*p* = 0.33) Symptom burden (ESAS) showed significant time effects for fatigue (*p* = 0.02), nausea (*p* = 0.009), and well-being/malaise (*p* = 0.02), whereas pain, depression, anxiety, appetite loss, shortness of breath, and drowsiness did not change significantly (Figure 1). Body weight was consistent across assessments: mean weight did not change significantly from baseline to T2 and the prevalence of ≥5% involuntary weight loss from baseline was 5.5% (3/55) at T1 and 23.6% (13/55) at T2 among patients with paired weights (Figure 2). Handgrip strength remained stable from baseline to T2 in both hands (right: *p* = 0.09; left: *p* = 0.20; Figure 3). No significant changes in biochemical or bioelectrical impedance parameters were observed over time (T0-T2; Figures 4A,B). Adherence to anticancer therapy was high among patients completing the nutritional plan (*n* = 70): 32/70 (46%) completed the planned regimen while 24/70 (34%) were still on treatment at data cut-off.

## 4 Discussion

This pilot study evaluated the feasibility and preliminary impact of an early nutritional screening and intervention pathway in patients with newly diagnosed head and neck and upper gastrointestinal cancers, two populations at particularly high risk of malnutrition due to tumor burden, treatment-related toxicities, and systemic inflammation. Despite strong recommendations from international guidelines supporting early nutritional screening in oncology patients (7–12), implementation in routine clinical practice remains suboptimal. A growing body of evidence has consistently documented a persistent gap between guideline recommendations and real-world practice in oncology nutrition, with nutritional screening often not integrated into standard workflows and referral to dietetic services remaining inconsistent (7, 10, 11). Recent implementation studies have shown that even when awareness exists among clinicians, structured nutritional assessment is frequently replaced by subjective judgment, resulting in delayed or incomplete identification of patients at nutritional risk (10, 12, 13). In this context, our findings provide additional real-world evidence of this implementation gap.

The overall enrolment rate in our study (14%) was substantially below the predefined feasibility threshold, indicating that the main barrier occurred at the level of referral rather than patient acceptance (1, 14). Once patients were enrolled in the structured pathway, adherence was high (91%), suggesting that patients are highly receptive to nutritional care when it is systematically offered. This pattern is consistent with recent quality improvement initiatives showing that structured or patient-centered screening strategies significantly increase referral rates compared with clinician-dependent approaches (30). Similar implementation studies have also demonstrated that embedding screening tools into routine clinical workflows is critical to improving nutritional referral rates in oncology settings (14, 15). Analysis of the 473 eligible but non-referred patients further supports this interpretation. The majority of these patients had preserved performance status (ECOG 0–1), and the predominance of patients with upper gastrointestinal tumors suggests that referral was largely driven by visible clinical deterioration rather than systematic screening. This reinforces the concept that reliance on subjective clinical assessment may underestimate early nutritional risk, a phenomenon previously reported in oncology populations where up to half of malnourished patients remain unidentified in the absence of structured screening systems (7, 10). Baseline assessments in the enrolled cohort confirmed a high prevalence of nutritional risk, with nearly half of patients classified as high risk according to MUST and showing functional impairment as reflected by reduced handgrip strength and phase angle values. Symptom burden was considerable even before therapy initiation, with fatigue, appetite loss, and malaise among the most frequent complaints, confirming that nutritional impairment and symptom distress coexist from the earliest disease stages. Despite most patients starting oncologic treatment during follow-up, body weight, handgrip strength, and overall quality of life remained stable, suggesting that early nutritional intervention may help prevent rapid deterioration. Significant time effects observed for fatigue, nausea, and malaise likely reflect treatment-related toxicity but also underline the importance of continuous nutritional monitoring to mitigate symptom burden. Taken together, these findings show that early nutritional screening and intervention are highly feasible and acceptable when properly integrated, providing a reproducible model for multidisciplinary care (31). These findings are consistent with previous studies demonstrating that functional and bioimpedance-derived parameters are sensitive indicators of early cancer-related nutritional decline (11, 12).

The choice of the Malnutrition Universal Screening Tool (MUST), rather than oncology-specific instruments such as the PG-SGA, warrants discussion. Although PG-SGA is widely regarded as the reference standard in oncology nutrition assessment due to its inclusion of symptom burden and metabolic factors, MUST was selected in the present study due to its simplicity and feasibility in routine outpatient practice, in line with the feasibility-oriented design of this pilot study. However, we acknowledge that MUST does not capture key nutrition-impact symptoms such as dysphagia, early satiety, or xerostomia, which are particularly relevant in head and neck and upper GI cancers. Therefore, the use of MUST may have underestimated the true prevalence and severity of malnutrition risk compared with more comprehensive tools, and this represents an important methodological limitation.

Regarding outcomes over time, body weight, functional parameters, and quality of life remained overall stable during follow-up, with no significant changes in bioelectrical impedance or biochemical markers. However, given the absence of a control group and the short observation period, no causal inference can be made regarding the effect of the nutritional intervention. The observed stability may reflect the natural clinical course in the early phase after diagnosis and initiation of oncological treatment rather than a direct effect of nutritional support. This limitation is inherent to the exploratory design and should be addressed in future controlled studies with longer follow-up.

Tumor heterogeneity also represents an important limitation of this study. Patients with head and neck and upper gastrointestinal cancers were analyzed within a single cohort despite substantial differences in pathophysiology, symptom burden, and treatment-related nutritional impact. For example, chemoradiotherapy in head and neck cancer is primarily associated with mucositis and dysphagia, whereas upper GI tumors are more frequently characterized by malabsorption, early satiety, and metabolic derangements. Although stratified descriptive analyses were provided, the sample size did not allow formal subgroup comparisons, limiting the ability to identify differential responses to the intervention.

Finally, several relevant clinical variables, including longitudinal treatment-related toxicities and longer-term oncological outcomes, could not be assessed due to the short follow-up duration (2 months). This precludes evaluation of clinically meaningful endpoints such as treatment completion, hospitalization rates, or survival outcomes. Future studies should adopt longer follow-up periods (at least 6–12 months) to better characterize the sustained impact of early nutritional intervention in this population.

In summary, this study demonstrates that early nutritional screening and intervention is highly feasible and well accepted by patients once implemented, but its effectiveness is strongly limited by low referral rates driven by the absence of standardized screening pathways. These findings highlight that the main barrier to optimal nutritional care in oncology is not patient compliance, but system-level implementation failure. Embedding objective nutritional screening tools into routine oncology workflows, potentially supported by automated electronic prompts or multidisciplinary care pathways, may represent a crucial strategy to bridge this gap and improve early identification and management of malnutrition in cancer patients.

### 4.1 Limitations

The present study has several limitations that should be acknowledged. First, its single-center design and relatively small sample size limit the generalizability of the findings to other oncology settings. Second, the heterogeneity of tumor types included (head and neck and multiple upper GI cancers) introduces clinical variability in nutritional risk profiles, treatment-related toxicities, and disease trajectories, which could not be fully accounted for due to limited statistical power for subgroup analyses. Third, the short follow-up period (2 months) does not allow assessment of longer-term clinically relevant outcomes such as treatment completion, hospitalization rates, postoperative complications, or survival. Fourth, the absence of a control group precludes any causal inference regarding the effect of the nutritional intervention on clinical, functional, or quality-of-life outcomes, which should therefore be interpreted as exploratory and hypothesis-generating. Fifth, several relevant variables, including detailed dietary intake, full longitudinal biochemical profiles, and extended bioelectrical impedance parameters, were available only in a subset of patients, reflecting real-world clinical practice but potentially introducing information bias. Finally, referral to the nutritional pathway was not standardized and relied on clinician judgment, and no formal qualitative assessment of barriers to referral among healthcare professionals was performed. This limits the ability to fully characterize the determinants of the observed low enrolment rate.

## 5 Conclusion

This pilot study demonstrates that early nutritional screening and intervention in patients with head and neck and upper gastrointestinal cancers is highly feasible and well accepted once patients are enrolled into a structured pathway. However, implementation at the population level remains limited by low and non-systematic referral rates, suggesting that the main barrier to effective nutritional care lies within oncology workflow organization rather than patient adherence. The findings highlight a critical implementation gap between guideline recommendations and routine clinical practice, emphasizing the need for standardized, systematic, and potentially automated nutritional screening strategies to ensure early identification of patients at risk of malnutrition.

Future multicenter studies with longer follow-up, inclusion of control groups, and integration of disease-specific nutritional assessment tools are warranted to confirm clinical benefits and optimize implementation strategies in real-world oncology care.

## Data Availability

The dataset used and analyzed during the current study is available from the corresponding author upon reasonable request. Requests to access the datasets should be directed to irene.azzali@irst.emr.it.
